# Serologic screening for viral infections among blood donors: a study in a blood bank in southern Brazil

**DOI:** 10.1590/1806-9282.20240452

**Published:** 2024-09-02

**Authors:** Gabriella Shinmi Belanda, Mariana Fardin, Thelma Larocca Skare, Claudia Alexandra Pontes Ivantes, Karla Braga Fávero, Paulo Tadeu Rodrigues Alemida, Mateus Oliveiro de Almeida, Renato Nisihara

**Affiliations:** 1 Mackenzie Evangelical School of Medicine of Paraná – Curitiba (PR), Brazil.; 2 Universidade Federal do Paraná, Department of Medical Clinic – Curitiba (PR), Brazil.; 3 Institute of Hematology, Hemobanco – Curitiba (PR), Brazil.

**Keywords:** Blood donors, Viral diseases, Viral hepatitis, HIV

## Abstract

**BACKGROUND:**

Routine screening for viral infections at blood donation is important to avoid transfusion-transmitted infections. It also offers an opportunity to detect an asymptomatic infection.

**OBJECTIVE:**

To study changes in serology positivity for viral infections (B and C hepatitis, HTLV-1/2, and HIV) at blood donation in a blood bank from Southern Brazil, comparing two periods of 5 years: the period from 2013 to 2017 with the period from 2018 to 2022. In addition, data on the donor fidelity rate during the studied period were sought.

**METHODS:**

Retrospective study using data from 2013 to 2022 from a single blood center electronic database from Curitiba, Southern Brazil.

**RESULTS:**

A significant drop in positive serology for all studied viruses was observed: highest in HIV (OR=0.39; 95% CI=0.27–0.57) and lowest in total anti HBc (0.56; 95 CI=0.50–0.63). Anti HBc serology became more commonly seen in women in the period of 2018–2022 when compared to men. No changes in the distribution of positive serology according to donors’ ages were observed. Loyalty rates had a median of 70%, with the lowest being 60% in 2013, while the highest was 73% in 2018 and 2022.

**CONCLUSION:**

A significant reduction in discarded blood bags due to viral serology was observed when the period of 2013–2017 was compared to 2018–2022 on this blood bank; the highest reduction was observed in HIV serology and the lowest in HBc serology, which became more common in women in the second period. High rates of donor fidelity were observed during the period studied.

## INTRODUCTION

While blood transfusion may be a lifesaving or health-improving medical intervention, it also carries some risks and complications^1^. Transfusion-transmitted viral infections such as hepatitis C and B, infection by human immunodeficiency virus (HIV), and human T cell lymphotropic virus (HTLV) 1/2 are some of them^1^. To ensure a safe blood transfusion, screening for viral infections is routinely included among the protective measures used at blood banks.

Hepatitis B and C are major causes of chronic hepatic diseases, liver cirrhosis, and hepatocarcinoma^2^, as well as several extrahepatic manifestations such as mixed cryoglobulinemia, lymphoproliferative disorders, renal disease, insulin resistance, type 2 diabetes, and rheumatic manifestations^3,4^. From 1999 to 2020, approximately 689,933 new cases of viral hepatitis were registered in Brazil, 254,389 (36.9%) cases of hepatitis B, and 262,815 (38.1%) of hepatitis C^2^. In this same time period, 78,642 deaths from viral hepatitis were recorded (21.3% from hepatitis B and 76.2% from hepatitis C)^1^. Fortunately, hepatitis B vaccination and effective treatment for hepatitis C have reduced the burden of these two infections in the general population^5^. In Brazil, hepatitis B vaccination was initially introduced in the National Immunization Calendar in the 1990s and was intended for all children in their first year of life; in 2016, the coverage was expanded to include all individuals, irrespective of age^6^. Although there is no effective vaccination for hepatitis C, treatment with direct-acting antiviral agents has resulted in high rates of sustained virologic response^5^. interrupting the sequence of transmission. Considering this, it is possible to hypothesize that, among blood donors, the number of individuals who are positive for hepatitis B and C has dropped recently, reducing the number of blood bags discarded because of these infections.

HTLV-1 is considered the most oncogenic human retrovirus pathogen^7^; it is associated with the occurrence of at least two severe diseases: adult T cell leukemia-lymphoma^8^ and HTLV-1-associated myelopathy, a fatal disease also known as tropical spastic paraparesis^9^. This infection has also been associated with uveitis, autoimmune thyroiditis, myositis, arthritis, and Sjogren's syndrome^10^. Differently from other viral blood transmitted infections, HTLV-1 results in the transference of infected lymphocytes rather than cell-free viral particles^11^. Its structure is very similar to that of HIV, but it differs in that it does not induce T cell death but rather cell proliferation and transformation^12^. The frequency of HTLV infections in Brazil ranges from 0.01 to 1.35% in the general population, depending on the geographical area and the presence of behavioral risk factors^13^. It is more frequent in women, with lower levels of education^14^. In Brazil, blood products have been obligatorily screened for HTLV infections since 1993^14^.

Regarding HIV, from 2007 to July 2021, 381,793 infections were registered in Brazil: 69.8% in men and 30.2% in women^14^. According to the Ministry of Health, there was a decrease of 35.7% in the detection rate from 2012 to 2020, but underreported rates during the COVID-19 pandemic may be responsible for this reduction, especially in 2020^14^.

The purpose of this study was to investigate the prevalence of seropositivity for viral infections – HIV 1/2, HTLV1/2; hepatitis B and C – in blood donors from a southern Brazilian blood bank, comparing rates of positive serology from 2013 to 2017 with 2018 to 2022, with the objective of accounting for the demographic changes that may have occurred during this time period. In addition, data on the donor loyalty rate during the studied period were sought.

## METHODS

### Ethical issues

This study was approved by the local Research Ethics Committee under protocol number 5.902.331, effective February 2023.

### Design study and data collection

A retrospective study was conducted using data from 2013 to 2022 from a single blood center electronic database in Curitiba, southern Brazil.

The study took into consideration the number of blood donations per year, donor loyalty rates in the period studied, the number of annual donations by sex, and the total number of blood donors rejected annually due to positive viral hepatitis, HTLV-1, and HIV serologies from January 2013 to December 2022. The study period was divided into two groups, the first 5 years (2013–2017) were compared to the last five years (2018–2022).

The National Health Surveillance Agency (ANVISA) standards were followed at this blood bank. Validated commercial kits that detect antibodies or antigens by immunoenzymatic methods (Chemiluminescence) using automated procedures (Alinity, Abbott, Lake Forest, USA) were used to detect hepatitis B (anti-HBc and HBsAg), hepatitis C (anti-HCV), HIV (anti-HIV-I, anti-HIV-II, and HIV antigen testing p24), and HTLV (anti-HTLV-1 and anti-HTLV-2).

According to the recommendation of ANVISA, when the initial sample taken at the time of donation was positive, it was considered a rejected donation by positive serology, regardless of subsequent confirmatory testing^15^.

### Statistical analysis

Data was expressed in absolute numbers, and frequencies were expressed in percentages. Comparison analysis was performed using the χ^2^ test using the software Graph Pad Prism version 6.0 (GraphPad Software Inc., La Jolla, CA, USA). p-values less than 0.01 were considered significant.

## RESULTS

Approximately 264,922 blood donations were registered between 2013 and 2022, with 46.0% from women and 53.9% from men. During this time period, 216 (0.082%) blood bags were rejected due to positive serology for hepatitis C; 106 (0.040%) due to positive HBsAg; 1485 (0.561%) due to positive total anti-HBc; 71 (0.027%) due to positive HTLV; and 136 (0.051%) due to positive HIV.

Donor loyalty rates in the period had a median of 70%, with the lowest in 2013 (64%) and the highest in 2018 and 2022 (73% in both).

**Figure 1:**
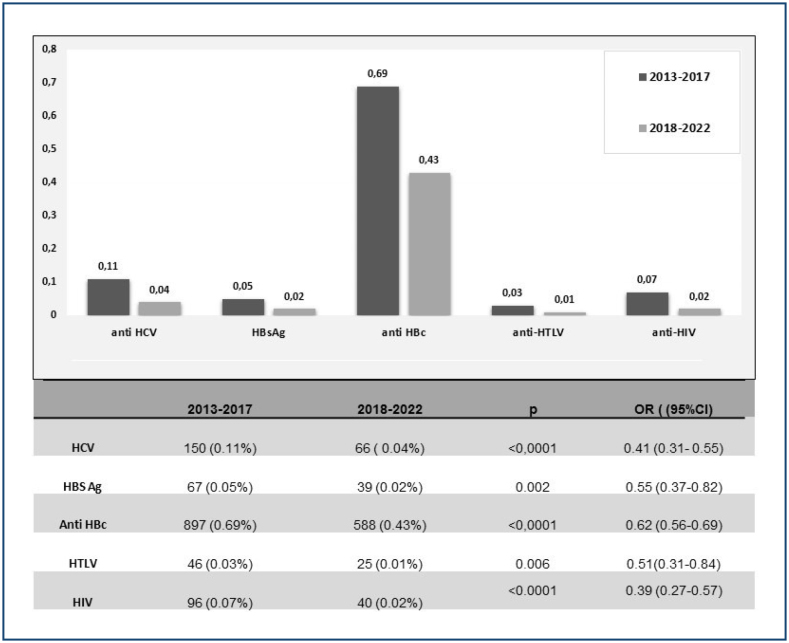
Comparison of positive viral serology between 2013–2017 and 2018–2022.

Figure 1 shows the results of the first 5-year time period (2013–2017) compared to the last 5-year time period (2018–2022). A significant reduction in positive serology for all studied viruses was found, with HIV showing the greatest reduction and total anti-HBc serology the lowest.

The comparison of positive serology by sex for these two time periods is shown in Table 1. In both time periods, women had a slightly higher positive rate in total anti-HBc than men; in the years 2018–2022, the proportion of positivity increased in women and decreased in men significantly. The proportion of changes in positive HIV serology was similar in both sexes.

**Table 1:** Comparison of discharged bags according to viral serology by sex during the periods of 2013–2017 and 2018–2022.

		Women	Men	p	OR (95%CI)
HCV	2013–2017	40.0%	60.0%	0.65	–
	2018–2022	43.3%	56.6%		
HBs Ag	2013–2017	43.2%	56.7%	0.77	–
	2018–2022	46.1%	53.8%		
Total anti-HBc	2013–2017	51.0%	48.9%	0.004	OR=1.36 (1.10–1.67)
	2018–2022	58.6%	41.3%		
HTLV	2013–2017	48.6%	51.3%	0.72	
	2018–2022	50.8%	49.1%		
HIV	2013–2017	52.7%	47.2%	0.35	
	2018–2022	48.8%	51.1%		

The distribution of rejection bags based on positive serology and age is shown in Table 2.

**Table 2:** Comparison of discharged bags due to viral serology according to donors’ age during the periods of 2013–2017 and 2018–2022.

		<20 years	20–39 years	40–59 years	≥60 years	p
HCV	2013–2017	3.8%	53.0%	41.5%	1.5%	0.42
	2018–2022	1.1%	61.6%	34.8%	2.3%	
HBs Ag	2013–2017	4.4%	55.2%	32.8%	7.4%	0.14
	2018–2022	0	56.4%	43.5%	0	
Anti-HBc	2013–2017	2.2%	50.1%	45.3%	2.2%	<0.0001
	2018–2022	0.8%	37.2%	59.1%	2.7%	
HTLV	2013–2017	25.3%	31.3%	27.3%	16.0%	0.95
	2018–2022	24.1%	34.4%	26.7%	14.6%	
HIV	2013–2017	27.1%	28.5%	26.6%	17.7%	0.62
	2018–2022	23.8%	32.0%	28.5%	15.6%	

## DISCUSSION

Screening for viral infections during blood donation not only reduces the risk of transmission but also offers an opportunity to detect the carrier of an asymptomatic disease and establish proper treatment. The prevalence of blood bags rejected by these infections may provide some perspective on how these infections affect the local region. Although blood donors are usually healthier than the general population and most positive serological tests detected at blood donation are in asymptomatic or unknown carriers, and in a lower proportion than the overall population, the change in serological test positivity over time may show a trend in the prevalence of infections in a given geographical region. In this study, there was a significant reduction in the prevalence of all studied viral infections from the first five studied years (2013–2018) compared to the second five studied years (2019–2022). The most commonly positive serology was for total anti-HBc, which also had the lowest serology reduction between the two studied periods.

The first viral antigen identified in the blood after an individual has an acute-phase infection by the hepatitis B virus is hepatitis B surface antigen (HBsAg), which remains positive even in the chronic phase, but becomes undetectable after the virus is cleared. Therefore, HBsAg is the infection marker used to detect the virus^16^. The core particle (HBc) encloses the HBV genome; the anti-HBc IgM is produced when the host clears the HBsAg by producing HBsAg antibodies. Isolated total anti-HBc (with negative HBsAg and anti-HBs) is the only marker of HBV infection when the host has undetectable levels of HBsAg, but has not yet developed anti-HBs. Blood donors are infectious at this stage; therefore, it is necessary to measure total anti-HBc during screening^16-18^. Chronic HBV infection is associated with IgG anti-HBc antibodies. Anti-HBc antibodies are also useful in detecting occult HBV infection, which occurs when there is low-level HBV DNA in the serum, hepatic tissue, or immune cells of a patient with serological markers of the previous infection (anti-HBc or anti-HBs positive) and the absence of serum HBsAg. Therefore, a positive anti-HBc antibody is a key marker of an occult HBV infection^17-20^. A significant difference between the positivity of HBsAg and anti-HBc was found in this study, emphasizing the importance of undertaking both serologic tests.

In most blood banks, including the one where this study was conducted, screening is done with HBsAg and anti-HBc total (IgM+IgG), and the latter test is useful to identify previous and current HBV infection^15^. Curiously, the proportion of women detected by anti-HBc but not by HBsAg increased during the second studied time period (2018–2022). Fasola et al.^16^ found no sex differences in anti-HBc positive blood donors in a Nigerian Blood Bank, but this prevalence was higher in individuals over the age of 36. In our study, almost all detected infections occurred in donors aged 20–59 years during the two studied periods. In a study carried out at the same blood bank in the period ranging from January 2003 to December 2012, of the total number of donations, the serological testing with the highest positivity was anti-HBc (2.7% of discards), HIV (0.9%), hepatitis C (0.8%), HBsAg (0.3%), and HTLV (0.2%)^21^. Data from the Brazilian Ministry of Health showed that between 2011 and 2019, hepatitis B detection rates in Brazil decreased by 20.7%, from 8.5 to 6.7 cases per 100,000 inhabitants. In 2021, the detection rate dropped to 3.4 cases per 100,000 inhabitants, the lowest recorded in the historical series^2^.

Among the studied serological tests, HIV positivity showed the greatest decline. Interestingly, the proportion of HIV seropositive donors was similar in both sexes, despite the fact that the infection is more frequent among men in the general population^2^. Concerning HIV testing, it has been observed that, in Brazil, some individuals seek the blood bank in order to have the HIV test done, due to unfavorable perceptions of free public Volunteer Testing Centers^22^. This may have affected the results, but despite this possibility, HIV testing has shown the greatest reduction among the tested viral serologies.

When the two study periods were compared, HTLV positivity also decreased, but the sex and age distribution profiles remained unchanged.

In our study, we observed a high rate of fidelity among blood donors (above 70%). Blood donor fidelity is crucial to reducing positivity rates in serological tests^23^. Regular donors undergo frequent and rigorous screening, increasing the likelihood of identifying and ruling out possible infections. Additionally, continued education about risk behaviors and health care reinforces the responsibility of loyal donors^23^. In this way, fidelity not only ensures a constant supply of safe blood, but also contributes to the integrity of the donation system.

This study has some limitations inherent to its design. The study design, using a database, did not allow access to donor clinical information. All positive tests for the first test were confirmed, but unfortunately, we do not have access to confirmatory test data.

In conclusion, a significant reduction in discarded blood bags due to viral serology was observed between 2013–2017 and 2018–2022 on this blood bank; the highest reduction was observed in HIV serology and the lowest in HBc serology, which became more common in women during the two observed periods. High rates of donor fidelity were observed during the period studied.

## ETHICS APPROVAL

All procedures performed in studies involving human participants were in accordance with the ethical standards of the institutional research committee and with the 1964 Helsinki declaration and its later amendments or comparable ethical standards. This study was approved by the Committee of Ethics in Research from Institution under protocol number 5.902.331.

## CONSENT TO PARTICIPATE

All participants signed an informed consent.

## CONSENT FOR PUBLICATION

Yes.

## TRANSPARENCY DECLARATION

The authors affirm that this manuscript is an honest, accurate, and transparent account of the study being reported; that no important aspects of the study have been omitted; and that any discrepancies from the study as planned (and, if relevant, registered) have been explained.
